# Targeting the cell cycle for cancer therapy

**DOI:** 10.1038/sj.bjc.6600458

**Published:** 2002-07-02

**Authors:** A Carnero

**Affiliations:** Experimental Therapeutics Programme, Centro Nacional de Investigaciones Oncologicas (CNIO), c/Melchor Fernandez Almagro no. 3, 28029 Madrid, Spain

**Keywords:** cell cycle, CDK inhibitors, cancer therapy

## Abstract

Most if not all neoplasias show a directly or indirectly deregulated cell cycle. Targeting its regulatory molecules, the cyclin-dependent kinases, as a therapeutic mode to develop new anticancer drugs, is being currently explored in both academia and pharmaceutical companies. The development of new compounds is being focused on the many features of the cell cycle with promising preclinical data in most fields. Moreover, a few compounds have entered clinical trials with excellent results maintaining the high hopes. Thus, although too early to provide a cell cycle target based new commercial drug, there is no doubt that it will be an excellent source of new anticancer compounds.

*British Journal of Cancer* (2002) **87**, 129–133. doi:10.1038/sj.bjc.6600458 www.bjcancer.com

© 2002 Cancer Research UK

## 

Mammalian cell division is timely regulated by a family of protein kinase holoenzymes, the cyclin-dependent kinases (CDKs) and their heterodimeric cyclin partners. Regulation of CDK activity occurs at multiple levels, including cyclin synthesis and degradation, phospho- and dephosphorylation, CDK inhibitor (CKI) protein synthesis, binding and degradation, and subcellular localisation (Pines, 1995; Harper, 1997; Cerutti and Simanis, 2000). Orderly progression through the cell cycle involves coordinated activation of the CDK protein by binding to the cyclin partner (Table 1

**Table 1:**
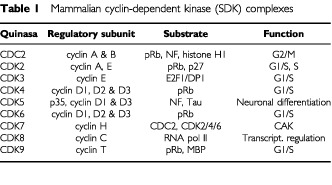
Mammalian cyclin-dependent kinase (SDK) complexes

). Different CDK-cyclin complexes operate during different phases of the cell cycle. The activation also requires the presence of CDK-activating kinase (CAK) that phosphorylates CDK subunits at residues Thr 160/161, and dephosphorylates residues Thr 14 and Tyr 15 by CDC25 phosphatase (Clarke, 1995; Draetta and Eckstein, 1997). Active CDK-cyclin complexes phosphorylate target substrates, including members of the ‘pocket protein’ family (the product of the retinoblastoma susceptibility gene, pRb, and the related p107 and p130 proteins) (Grana *et al*, 1998).

Endogenous inhibition of CDKs also occurs by two families of regulatory proteins induced under antimitogenic stimuli: the INK4 family, comprising p16^INK4a^, p15^INK4b^, p18^INK4c^ and p19^INK4d^, which contains conserved ankyrin motifs and specifically inhibits CDK4 and CDK6 (Carnero and Hannon, 1998). The CIP/KIP family includes p21^cip1/waf1^, p27^kip1^ and p57^kip2^, which share a broader range of inhibition and act in a concentration-dependent manner (Hengst and Reed, 1998). All CKIs cause G1 arrest when overexpressed in cells by association and inhibition of the CDKs. INK4 proteins dissociate the cyclinD/CDK complexes and redistributes the CIP/KIP proteins to CDK2 producing a double inhibition. At low concentration, CIP/KIP family proteins enhance CDK4 association to cyclin D increasing the activity of the complex. At high concentrations they inhibit the kinase activity, presumably by increasing the stechiometry in the CDK complexes (Sherr and Roberts, 1999).

Proper regulation of CDK activity is essential for the ordered execution of the processes controlling cell-growth, complete DNA replication and mitotic distribution of chromosomes to daughter cells. To ensure this, surveillance mechanisms function as checkpoints to control cell-cycle progression (Nurse, 1997). These checkpoints ensure that growth promoting or inhibiting signals transmit their effects on cell cycle progression by modulating CDK activity. After the proliferative stimuli (growth factors and oncogenes) inducing cell proliferation, a first checkpoint (the Restriction point) at late G1 integrates both positive and negative external and internal signals before the cell commits itself to another round of DNA replication. More specifically, this sensitive period includes the mid-to-late G1 phase culminating at the Restriction point (or R point), and the onset of DNA replication. In mammalian cells the R point is regulated mainly by the CDKs bound to the D type of cyclins (Draetta, 1994; Sherr, 1994). The G1 checkpoint ensures proper phosphorylation of the pRb protein. Phosphorylated pRb releases E2F transcription factors that are bound to DP1 forming the transcriptionally active heterodimer E2F-DP1 (Weinberg, 1995; Muller and Helin, 2000). This results in the expression of proteins (including cyclins A and E) necessary to initiate and complete DNA synthesis. Consistent with its role in the cell cycle, several E2F family member genes have been shown to function as oncogenes in culture. Removal of sequences involved in regulation by pRb, or inclusion of DP1, increases the oncogenic potential of E2F1. Also, a role for the E2F1 gene as a tumour suppressor has recently been established through the generation of mice lacking a functional E2F1 allele. Another important checkpoint occurs at the G2/M transition which ensures proper chromosome segregation to the daughter cells (Ohi and Gould, 1999). This checkpoint is controlled by CDK1 (CDC2) bound to cyclins B and A. Although it appears that the oncogenic defects may target any major transition or checkpoint of the cell cycle, the step strikingly deregulated most frequently is G1 to S transition.

The vast majority of human neoplasias have abnormalities in one or more of its cell cycle components, due to overexpression of positive regulators of CDK function and/or a decrease in the negative regulators of CDK function resulting in hyper activation of CDKs (Table 2

**Table 2:**
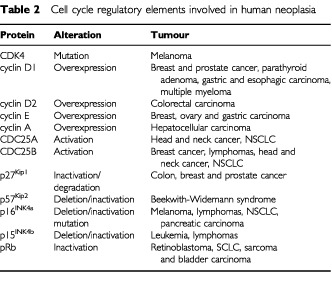
Cell cycle regulatory elements involved in human neoplasia

). However, the involvement of the cell cycle mechanism in tumorigenesis is not restricted to the direct deregulation of one or more of its components. Tumorigenic properties of many oncogenes also rely on their ability to deregulate the cell cycle machinery (Hanahan and Weinberg, 2000).

Myc is a positive regulator of G1-specific CDKs in particular cycE/CDK2 complexes. Myc acts through at least three different pathways which can enhance CDK function; (1) Functional inactivation of the CDK inhibitor p27^kip1^ and probably also p21^cip1/waf1^ and p57^kip2^; (2) induction of the CDK-activating phosphatase CDC25A and (3) deregulation of cyclin E expression (Amati *et al*, 1998).

Ras transduces mitogenic stimuli in response to tyrosine-kinase receptors, and its function is required in G1 for passage through the R-point (Malumbres and Pellicer, 1998; Crespo and Leon, 2000). Ras activity is required for the phosphorylation of pRb in response to mitogenic signalling, and functional inactivation of Ras induces G1 arrest in pRb-positive but not in pRb-negative cells. The mitogenic signal mediated by Ras and Raf may act through the induction of cyclin D1 and/or the degradation of p27^kip1^. Activated Ras protein, or its effector Raf, maintain a constitutive mitogenic signal mimicking a constitutive activation of Tyr kinase receptors. Other oncogenes such as *Erb*2, *src*, v-*sis*, may act, at least partially, by similar mechanisms.

Abnormalities in the p53 tumour suppressor gene are the most frequent molecular events in human and animal neoplasia. p53 is a critical nodal point of converging pathways from diverse cellular insults which can elicit coordinated cellular response that result in adaptation to the insult (Prives and Hall, 1999; Bates and Vousden, 1996). p53 functions as a transcription factor inducing MDM2, Bax or IGF-BP3, and represses *myc*, *fos* or *Bcl*2. p53 also induces genes that directly interfere with the cell cycle such as p21waf1, 14-3-3σ or GADD45. Either, 14-3-3σ or GADD45 overexpression arrests cells at G2/M by inhibiting cycB/CDC2 activity. In cells containing wild type p53, γ-irradiation causes G1 arrests due to a p53-dependent activation of p21^cip1/waf1^. Furthermore, other oncogenes or tumour suppressors function through direct or indirect derregulation of p53 or pRb pathways such as MDM2, p14/19^ARF^, p33^ING1^, BMI-1, BRCA1, or viral oncogenes as HPV E6/E7, E1a, E1b or SV40 T antigen.

Given that the ultimate goal of cancer research is to find the complete cure for as many tumour types as possible, the identification of cell cycle targets may decisively influence the outcome of therapy.

## APPROACHES TO CDK INHIBITION

Because of the complex nature of its regulation, pharmacological modulation of CDK function was thought to be improbable. However, with the advent of newer biological probes and techniques, modulating CDK activity can be approached via multiple modes for therapeutic intervention.

### Chemical inhibitors of CDK

CDKs are small ser/thr kinases that display 11 common subdomains shared by all protein kinases. The ATP-binding site, located in a deep cleft between the two lobes of the protein, contains the catalytic residues, which are conserved across eukaryotic protein kinases (Pavletich, 1999). Small molecules that interact specifically with the ATP-binding site of CDKs represent the most immediate opportunity to allow pharmacological design. A group of compounds that occupy the ATP-binding pocket of the enzyme and are competitive with the ATP have been characterized (Meijer *et al*, 1999; Mani *et al*, 2000; Fischer and Lane, 2000; Senderowicz and Sausville, 2000). Chemical CDK inhibitors can be subdivided into the following eight families: purine derivatives (including 6-DMAP, olomucine, roscovitine and purvanolol), butyrolactone I, flavopiridols, staurosporine and derivatives, toyocamycin, 9-hydroxyellypticine, polysulphates (including suramin) and paullones, although new ones are constantly being discovered. Compounds such as staurosporine and their derivatives, suramin and 6-DMAP are relatively non-specific kinase inhibitors. Compounds much more selective for CDK inhibition versus other kinases include flavopiridol that inhibits all CDKs, however, butyrolactone I, olomucine and roscovitine, purvanolol and paullone derivatives are relatively selective for CDK1(CDC2) and CDK2, but spare CDK4 and CDK6. The antiproliferative effects of these compounds on the growth of several human cell lines has been well documented (Sausville *et al*, 1999; Meijer *et al*, 1999). The effects *in vivo* of these compounds paralleled the *in vitro* efficiency and were further confirmed with the use of dominant negatives, the overexpression of natural CKIs and the microinjection of inactivating antibodies or antisense technologies (van den Heuvel and Harlow, 1993).

Some of these CDK inhibitors have been piloted in the clinic and the first data from these clinical trials are available (Senderowicz *et al*, 1998; Stadler *et al*, 2000; Schwartz *et al*, 2001). Flavopiridol, the first CDK modulator tested in clinical trials, demonstrated interesting clinical features: cell cycle block at G1 and G2 (consistent with its inhibition of CDK2 and CDK1), induction of apoptosis, promotion of differentiation, inhibition of angiogenic processes and modulation of transcriptional events. Besides the direct effects flavopiridol depletes cyclin D1 and D3 by transcriptional repression (Senderowicz, 2001). This may be a consequence of the direct inhibition of CDK9-cyclinT (also known as P-TEF, positive transcription elongation factor). Interestingly, in contrast with other CDKs, flavopiridol was not competitive with ATP (Chao *et al*, 2000). P-TEF is a required cellular cofactor for the human HIV transactivator Tat. Flavopiridol blocked Tat transactivation and blocked HIV-1 replication *in vitro* assays. This biochemical effect indicates that flavopiridol should be tested in HIV malignancies including HIV-lymphomas. Initial clinical trials with infusional flavopiridol demonstrated activity in some patients with a variety of tumour types, including non-Hodgkin's lymphomas, renal, colon and prostate cancers. The second CDK modulator tested in clinical trials is the staurosporine derivative UCN-01. UCN-01 also blocks cell cycle progression and promotes apoptosis. Moreover, UCN-01 may abrogate checkpoints induced by genotoxic stress due to inhibition of Chk-1 kinase. UCN-01 showed a long plasma half-life (approximately 600 h) due to binding to the alpha-1-acid-glycoprotein. Clinical activity was detected against melanoma, lung cancer and non-Hodgkin's lymphoma.

Phase II trials with these compounds in other schedules or in combination with standard chemotherapic agents are ongoing.

### Protein- and peptide-based inhibitors

CKIs combined with adenovirus vectors as vehicles for delivery and expression are a powerful approach to examine therapeutic applications of CDK inhibition. Introduction of p16^INK4a^ in tumour cells with functional pRb induces growth arrest of the cells at G1 phase (Jin *et al*, 1995; Craig *et al*, 1998). Similar results have been obtained with pRb and other members of the INK4 family (Schreiber *et al*, 1999). Adenovirus expressing either p21^cip1/waf1^ or p27^kip1^ in cancer cell lines also demonstrated both *in vitro* and *in vivo* growth inhibition (Chen *et al*, 1996; Craig *et al*, 1997).

Based on this, the strategy of using small peptides that mimic the effects of endogenous CDK inhibitors is being developed.

Several carriers have been tested that introduce peptides into cells. When a 16-amino acid transmembrane carrier segment derived from the *Drosophila* antenappedia protein was linked to the third ankyrin repeat of the p16^INK4a^ protein and inserted into cells, Rb-dependent G_1_ arrest was observed (Fahraeus *et al*, 1996). In a breast-derived cell line, the chimera containing antennapedia peptide and the carboxyl-terminal residue of p21^cip1/waf1^, had higher specificity for cdk4/cyclin D than for cdk2/cyclin E and arrested the cells in G_1_ phase (Ball *et al*, 1997). In contrast, *in vitro,* the chimera containing amino-terminal peptides of p21^cip1/waf1^, inhibited both cdk1 and cdk2, and cells were arrested in all phases of the cell cycle (Bonfanti *et al*, 1997).

Chen *et al* (1999) have shown that 8-amino acid peptides derived from the putative cyclin-cdk2-binding region of p21^cip1/waf1^ and E2F1 linked to N-terminal residues derived from human immunodeficiency virus Tat protein or antennapedia protein can block cells in S phase. This effect was associated with a loss of cdk2 activity. Although all of the cells tested with these chimeras showed clear evidence of G_1_/S-phase arrest, immortalized/transformed cells were more prone to apoptotic cell death.

In another approach, a 20-amino acid peptide, identified by use of a combinatorial library, specifically binds cdk2 and inhibits its activity at low nanomolar concentrations *in vitro* (Colas *et al*, 1996). This peptide could act by blocking the interaction of the catalytic subunit with substrates or cyclin cofactors.

### Altering regulatory pathways

The depletion of the cyclin partner leads to CDK activity inhibition. Depletion of cyclin D1 from tumour cell lines with antisense fragments induced antiproliferative effects that were synergistic with other drugs (Schrump *et al*, 1996; Kornmann *et al*, 1998). Inhibition of either cyclin A or E synthesis or activity through microinjection of plasmids encoding antisense cyclin cDNA or affinity-purified anti-cyclin antibodies during G1 phase inhibited DNA synthesis, providing a basis for the use of this strategy as a therapeutic approach.

Apart from the use of antisense technologies to deplete the tumour cells from specific cyclins and/or CDKs, several compounds can inhibit tumour progression by modulating the levels of cell cycle proteins. In breast carcinoma cells, antiestrogens and retinoids inhibit the expression of cyclin D and other cell cycle related proteins inhibiting CDK activity. In some systems rapamycin treatment was associated with a decline in cyclin D1 and prevents IL-2-stimulated degradation of p27^kip1^. Cyclotoxic effects of flavopiridol may be also potentiated by an induced decrease in the stability of the mRNA of the antiapoptotic protein Bcl2. Other molecules, such as lovastatin, block cells at G1 concomitant with p21^cip1/waf1^ and p27^kip1^ induction and CDK inactivation. Butirate, a differentiating agent, suppresses the proliferation of tumour cells correlating with an increase in p21^cip1/waf1^ and a decrease in pRb phosphorylation.

Although it seems unclear that the growth inhibitory effects of these compounds are solely due to these modulating effects, they may contribute to the overall antiproliferative effects observed in preclinical studies.

### CDC25

CDC25 is a dual-specificity phosphatase, which removes inhibitory phosphorylations on Thr14 and Tyr15 residues on the ATP anchor motif of CDKs, activating the kinase. CDC25 has, therefore, been involved in cell transformation and tumorigenesis, checkpoint control and apoptosis (Draetta and Eckstein, 1997). Inhibition of CDC25A activity in HeLa cells using antibodies which inhibited cell division, resulted in the accumulation of cells with mitotic-like phenotype and death (Galaktionov and Beach, 1991). Inhibition of CDC25B using antisense approach lead to delay in S phase with subsequent antiproliferative effects (Garner-Hamrick and Fisher, 1998). Based on this information, the CDC25 family has been the subject of a screen for inhibitory compounds.

A number of metal anions are specific inhibitors of PTPases. Vanadate and tungstate have similarities with the phosphate group and can bind to the active site covalently. Glyoma cells treated with some vanadate derivatives arrest at the G2/M transition coinciding with a hyper phosphorylation of CDK1 and a dramatic decrease of its kinase activity (Faure *et al*, 1995). Other inactivating compounds found, such as dephostatin or nitric oxide itself can deactivate PTPases, perhaps by oxidation of the catalytic cystein residue. Such a redox mechanism has been suggested as a general regulatory mechanism *in vivo*. There is a moderate amount of CDC25 inhibitory compounds (such as sulfircin, DNAcins, vitamin K derivatives, dysidiolide, alkyllysophospholypids or naphtoquinone analogues) that have shown anti-tumour activity. However, most of them are still in preclinical studies (Eckstein, 2000). A few of them (such as hexadecylphosphocholine) have been studied in clinical trials against skin tumours and lung carcinomas. The results were unsatisfactory due to side effects, but the SAR of these compounds continue.

### Modulation of proteasome machinery

Sequential turnover of certain cell cycle regulators, including cyclins and CKIs p21^cip1/waf1^ and p27^kip1^, are mediated by the 20S proteasome, which promotes proteolytic degradation through the ubiquitin/proteasome pathway. Increased turnover of these cyclins are associated with the loss of CDK activity. On the other hand, the inhibition of p21^cip1/waf1^ or p27^kip1^ specific degradation could induce CDK inhibition through accumulation of the CKI. However, although theoretically possible, a further complication lies in the net effect and/or specificity of modulating proteasomal pathways. Non-specific modulation may alter many signalling pathways making difficult the prediction of the final cellular effect.

## OTHER RELATED APPROACHES

### E2F and apoptosis

Apoptosis as a consequence of Rb inactivation is largely dependent upon wild-type p53 function. This finding is consistent with the observation that in many human cancers with Rb mutations, p53 is also inactivated. It is also a likely explanation why DNA tumour viruses such as SV40, adenovirus, and human papilloma virus (HPV) encode proteins that target both Rb and p53. Given that ectopic expression of E2F1 can induce p53-dependent apoptosis (Wu and Levine, 1994), it is likely that deregulation of E2F1 participates in this protective apoptotic response as a consequence of Rb inactivation. The loss of this Rb-E2F1 apoptotic pathway may well explain why mice lacking E2F1 are predisposed to cancer. This is also being used to promote tumour cell death. Adenovirus mediated overexpression of E2F1 induced cell death in gastric carcinoma both *in vitro* and in mouse models (Fueyo *et al*, 1998). The apoptotic effect was more potent in the presence of other cell cycle inhibitors such as olomucine or roscovitine.

Adenovirus expression of E2F enhances the anticancer effect of p53 in glyomas (Mitlianga *et al*, 2001). E2F1 also inhibits MDM2 expression in MDM2-overexpressing tumours by inducing apoptosis, presumably by restoring p53 activity (Yang *et al*, 1999). Adenovirus mediated E2F1 gene transfer efficiently induced apoptosis in melanoma cells. Interestingly, this effect was observed independently of p53 status (Dong *et al*, 1999). Similar effects were observed in head and neck, breast and ovarian carcinoma cell lines.

### G2/M Checkpoint

The Polo-like kinase 1 (Plk1) is a highly conserved mitotic serine/threonine kinase which is commonly overexpressed in cancer cell lines (Golsteyn *et al*, 1996; Yuan *et al*, 1997). Plk1 positively regulates mitotic progression by activating the CDC25C-CDK1 amplification loop and by regulating late mitotic events, primarily the ubiquitin-dependent proteolysis. Antisense against Plk1 specifically inhibits cell proliferation of cancer cells in cell culture and in the nude-mouse tumour model, but did not inhibit growth and viability of primary cells.

Defects in pathways essential for mitotic regulation are likely to be implicated in the cascade of events leading to aneuploidy and neoplasia. Exogenous overexpression of AIM-1 increases ploidy and aneuploidy in human cells (Tatsuka *et al*, 1998). Overexpression in colorectal tumour cell lines is thought to have a causal relationship with multinuclearity and increased ploidy. Errors in cytokinesis caused by AIM-1 overexpression is a major factor in the predisposition to cancer. On the other hand, Aur2 has also been implicated in oncogenesis, probably inducing defects in kinetochore function leading to chromosome instability and human tumours (Goepfert and Brinkley, 2000). Although further studies are needed to provide a clearer definition of how these kinetic proteins are linked and regulated in normal mitosis and cancer, a defective mitotic apparatus and centrosome number are central and causative in chromosome missegregation and cancer. These proteins may provide new molecular targets to develop G2/M acting compounds inhibiting unrestricted proliferation.

## CONCLUDING REMARKS

Whilst still being at the beginning of a new era in drug discovery, it is clear that the information that is accumulating concerning the basic mechanisms that govern the cell cycle offers new hope and promise for developing a novel class of future medicines that specifically target aberrant proliferation. In this respect, there can be little doubt of the value of targeting the cell cycle in drug discovery.
